# Exosomes Derived from Mesenchymal Stem Cells Suppress Angiogenesis by Down-Regulating VEGF Expression in Breast Cancer Cells

**DOI:** 10.1371/journal.pone.0084256

**Published:** 2013-12-31

**Authors:** Jong-Kuen Lee, Sae-Ra Park, Bong-Kwang Jung, Yoon-Kyung Jeon, Yeong-Shin Lee, Min-Kyoung Kim, Yong-Goo Kim, Ji-Young Jang, Chul-Woo Kim

**Affiliations:** 1 Tumor Immunity Medical Research Center, Cancer Research Institute, Seoul National University College of Medicine, Seoul, South Korea; 2 Department of Pathology, Seoul National University College of Medicine, Seoul, South Korea; University of Southern California, United States of America

## Abstract

Exosomes are small membrane vesicles released by a variety of cell types. Exosomes contain genetic materials, such as mRNAs and microRNAs (miRNAs), implying that they may play a pivotal role in cell-to-cell communication. Mesenchymal stem cells (MSCs), which potentially differentiate into multiple cell types, can migrate to the tumor sites and have been reported to exert complex effects on tumor progression. To elucidate the role of MSCs within the tumor microenvironment, previous studies have suggested various mechanisms such as immune modulation and secreted factors of MSCs. However, the paracrine effects of MSC-derived exosomes on the tumor microenvironment remain to be explored. The hypothesis of this study was that MSC-derived exosomes might reprogram tumor behavior by transferring their molecular contents. To test this hypothesis, exosomes from MSCs were isolated and characterized. MSC-derived exosomes exhibited different protein and RNA profiles compared with their donor cells and these vesicles could be internalized by breast cancer cells. The results demonstrated that MSC-derived exosomes significantly down-regulated the expression of vascular endothelial growth factor (VEGF) in tumor cells, which lead to inhibition of angiogenesis *in vitro* and *in vivo*. Additionally, miR-16, a miRNA known to target VEGF, was enriched in MSC-derived exosomes and it was partially responsible for the anti-angiogenic effect of MSC-derived exosomes. The collective results suggest that MSC-derived exosomes may serve as a significant mediator of cell-to-cell communication within the tumor microenvironment and suppress angiogenesis by transferring anti-angiogenic molecules.

## Introduction

Exosomes are small membrane vesicles that originate from multivesicular bodies and they are secreted by a variety of cell types. Initially, exosomes became of interest since they were suggested to play a role in antigen presentation [1]. It has been demonstrated that exosomes can be used as a cell-free vaccine with therapeutic effects in cancer [2]. More recently, the finding that exosomes shuttle genetic materials, such as mRNAs and microRNAs (miRNAs), has shed new light on the role of exosomes in cell-to-cell communication [3]. Such novel mechanisms of intercellular communication raise the possibility that the transfer of genetic information via exosomes might modulate cellular activities in recipient cells [4].

miRNAs are small non-coding RNAs that regulate gene expression post-transcriptionally by targeting mRNAs. Recent evidence has demonstrated that miRNAs play a crucial role in both physiological and pathological processes [5]. It has been shown that miRNA mutations or misexpression is associated with various human cancers and some miRNAs can function as oncogenes or tumor suppressors [6]. A set of miRNAs have been found in microvesicles (MVs) released from different cell types, such as human renal cancer stem cells [7] and tumor-associated macrophages [8]. Moreover, it has been recognized that circulating miRNAs probably shuttled by exosomes/MVs in cancer patients can serve as novel diagnostic markers [9], [10].

Mesenchymal stem cells (MSCs) are multipotent cells that differentiate into osteoblasts, chondrocytes and adipocytes as well as cells of other mesodermal lineages [11]. Due to the fact that they can be recruited at sites of inflammation and tissue repair, the role of MSCs in regenerative medicine and their potential use as tools for gene delivery have been extensively studied [12]. Over the last decade, previous studies have demonstrated that MSCs can also migrate to the tumor microenvironment. Even though there has been an intense interest in the role of MSCs in cancer progression, the relationship between MSCs and tumor cells remains open to debate. Several studies have suggested that MSCs contribute to tumor progression and metastasis [13], [14], [15], whereas other reports have shown that MSCs suppress tumor growth [16], [17]. This dichotomy may reflect the possible involvement of a myriad of mechanisms, such as immune modulation, direct cell contact, and soluble factors [18].

The formation of new blood capillary vessels through the process of angiogenesis is essential for the growth of cancer [19]. Tumors frequently overexpress pro-angiogenic factors, such as vascular endothelial growth factor (VEGF), for their progression [20]. Studies on the effects of MSCs on angiogenesis have yielded paradoxical results. Several studies reported that MSCs promote vasculogenesis [21], [22], whereas other groups showed that angiogenesis is inhibited by MSCs [23], [24]. However, the effects of MSC-derived exosomes on tumor angiogenesis remain relatively unexplored.

In this present study, we hypothesized that MSC-derived exosomes might play a significant role in the tumor microenvironment, in particular, in terms of tumor vasculature. To address this hypothesis, we isolated exosomes from MSCs and identified MSC-derived exosomes. We next investigated the paracrine effects of MSC-derived exosomes on tumor angiogenesis by treating tumor cells with MSC-derived exosomes. Interestingly, we found that MSC-derived exosomes inhibited angiogenesis by down-regulating VEGF expression in tumor cells *in vitro* and *in vivo*. In addition, our data suggested that miR-16 shuttled by MSC-derived exosomes was partially associated with the down-regulation of VEGF in tumor cells. To the best of our knowledge, this is the first paper to suggest that miRNAs delivered by MSC-derived exosomes may reprogram the tumor microenvironment.

## Materials and Methods

### Cell culture

Mouse bone marrow-derived MSCs (Invitrogen, Carlsbad, CA) were cultured in αMEM (Invitrogen, Carlsbad, CA) supplemented with 10% fetal bovine serum (Welgene, Seoul, Korea), 1% penicillin-streptomycin (GIBCO-BRL Life Technologies, Gaithersburg, MD), and 1% L-Glutamine (Invitrogen, Carlsbad, CA). Mouse breast cancer cell line (4T1) and mouse endothelial cell line (SVEC) were obtained from the American Type Culture Collection (Manassas, VA). These cell lines were cultured in DMEM (Welgene, Seoul, Korea) containing 10% fetal bovine serum (Welgene, Seoul, Korea) and 1% penicillin-streptomycin (GIBCO-BRL Life Technologies, Gaithersburg, MD). All cells were cultured at 37°C with 5% CO_2_.

### Isolation of exosomes

MSCs were cultured in αMEM supplemented with 10% fetal bovine serum, 1% penicillin-streptomycin, and 1% L-Glutamine previously centrifuged at 100,000 g overnight to eliminate pre-existing bovine-derived exosomes [25]. MSCs were cultured for 48 h and MSC-derived exosomes were isolated using ExoQuick-TC^TM^ (System Bioscience, Mountain View, CA) according to the manufacturer's protocol. In brief, cell culture supernatants were harvested and centrifuged at 3,000 g for 15 min to remove cells and cell debris. Two milliliters of ExoQuick-TC Exosome Precipitation Solution was added to 10 ml of the supernatants and the mixture was refrigerated overnight. Then, the mixture was centrifuged at 1,500 g for 30 min and the supernatants were aspirated. The residual solution was centrifuged at 1,500 g for 5 min and removed. The exosome pellet was resuspended in the appropriate buffer for protein or RNA analysis.

### Western blotting

For Western blot analysis, proteins in cells or exosomes were extracted using a PRO-PREP™ kit (iNtRON Biotechnology, Seoul, Korea) and the concentrations of proteins were determined by a BCA protein assay kit (Pierce, Rockford, IL). A total of 50 μg of protein from cells or exosomes was separated on 10% SDS-PAGE and transferred to PVDF membranes (Bio-Rad, Hercules, CA). The blots were incubated with primary antibodies against CD63 (Santa Cruz Biotechnology, Inc., Santa Cruz, CA) and calnexin (Santa Cruz Biotechnology, Inc., Santa Cruz, CA) followed by incubation with HRP-tagged secondary antibodies (Santa Cruz Biotechnology, Inc., Santa Cruz, CA). The protein–antibody complexes were visualized using an enhanced chemiluminescence kit (Amersham, Arlington Heights, IL).

### Cellular uptake of MSC-derived exosomes

MSC-derived exosomes were labeled with PKH26 (Sigma-Aldrich, St. Louis, MO) as previously described [26] with minor modification. In brief, 2 μl of PKH26 was added to 25 μg of MSC-derived exosomes in a total of 1 ml of diluent and incubated for 20 min at room temperature. A mixture without exosomes was used as a negative control to examine any carryover of PKH26 dye. One milliliter of 1% bovine serum albumin (BSA) was added to stop labeling and the mixture was added into 18 ml of phosphate buffered saline (PBS) and centrifuged at 100,000 g for 2 h at 4°C. The supernatant was removed and the pellet was resuspended in 20 ml of PBS and then centrifuged at 100,000 g for 2 h at 4°C. The pellet containing PKH26-labeled exosomes was resuspended in 2 ml of DMEM. 4T1 cells were previously cultured to 60% confluency and the medium was replaced with DMEM containing PKH26-labeled exosomes and cells were incubated for 24 h at 37°C with 5% CO_2_. After incubation, cells were washed twice with PBS and fixed in 4% paraformaldehyde for 20 min at room temperature. The sample was washed twice with PBS and mounted with a coverslip using Vectashield mounting medium containing 4,′6′-diaminido-2-phenylindole (DAPI; Vector Laboratories, Burlingame, CA). Cellular uptake of MSC-derived exosomes was observed under the confocal laser microscopy (Zeiss, Oberkochen, Germany).

### Enzyme-linked immunosorbent assay

Approximately, 7×10^5^ 4T1 cells were seeded in a 6-well plate and incubated with various concentrations of MSC-derived exosomes (25 μg/ml, 50 μg/ml, and 100 μg/ml) or carrier control (PBS) for 24 h. The supernatants were harvested and the amount of secreted VEGF was quantified using a commercial enzyme-linked immunosorbent assay kit (R&D Systems, Minneapolis, MN) according to the manufacturer's recommended protocol.

### RNA extraction and analysis

Total RNA was isolated from MSC-derived exosomes or other cells using TRI Reagent (MRC, Inc., Cincinnati, OH). RNA concentration was examined using a ND-1000 (NanoDrop Technologies, Wilmington, DE) and RNA content was analyzed using an Agilent 2100 Bioanalyzer (Agilent Technologies, Inc., Santa Clara, CA).

### mRNAs analysis by qRT-PCR

For each sample, 2 μg of RNA was treated with DNase I (Promega, Madison, WI) to eliminate residual genomic DNA and subjected to reverse transcription using a PrimeScript™ 1st Strand cDNA Synthesis Kit (Takara Bio, Shiga, Japan). qRT-PCR was performed using SYBR Premix Ex Taq (Takara Bio, Shiga, Japan) and an iCycler IQ thermocycler (Bio-Rad, Hercules, CA) according to the manufacturers' instructions. The following PCR primers were used: VEGF primers, forward: 5′-GAGCAGAAGTCCCATGAAGTGA-3′, reverse: 5′-CACAGGACGGCTTGAAGATGT-3′, VEGFR-1 primers, forward: 5′-GAGGAGGATGAGGGTGTCTATAGGT-3′, reverse: 5′-GTGATCAGCTCCAGGTTTGACTT-3′, GAPDH primers, forward: 5′-GGGCTGGCATTGCTCTCA-3′, reverse: 5′-TGCTGTAGCGTATTCATTG-3′.

### miRNAs analysis by qRT-PCR

For qRT-PCR, cDNA was generated from 250 μg of DNase I-treated RNA using a Mir-X miRNA First Strand Synthesis Kit (Takara Bio, Shiga, Japan). qRT-PCR was performed using a SYBR qRT-PCR Kit (Takara Bio, Shiga, Japan) and an iCycler IQ thermocycler (Bio-Rad, Hercules, CA) as described above. All reactions were performed in a 25 μl reaction volume in triplicate. Expression levels were obtained using threshold cycles (Ct) that were determined by the iCycler iQ Detection System software (Bio-Rad, Hercules, CA). Relative transcript quantities were calculated using the ΔΔCt method [27].

### Transfer of miRNAs

miRNA transfer experiment was performed as previously described [28]. Briefly, 7×10^5^ 4T1 cells were seeded in a 12-well plate prior to initiation of the experiment. After 1 day, 4T1 cells were co-incubated with MSC-derived exosomes (100 μg/ml) and α-amanitin (50 μg/ml) (Sigma-Aldrich, St. Louis, MO), a transcription inhibitor or α-amanitin alone to suppress transcriptional activation caused by exosomes. To eliminate any residual of MSC-derived exosomes, cells were washed twice with PBS and harvested at time after 0, 8, and 24 h. Total RNA from 4T1 cells was extracted and qRT-PCR for miR-16 was conducted according to the protocol described above. To analyze miR-16 transfer, we measured the difference in Ct values between α-amanitin-treated cells in the absence or in the presence of MSC-derived exosomes.

### Transfection of 4T1 cells with miRNA inhibitor

miR-16 inhibitor was purchased from Genepharma (Shanghai, China) and G-Fectin (Genolution Pharmaceuticals, Seoul, Korea) was used as the miR-16 inhibitor delivery reagent. Approximately, 7×10^5^ 4T1 cells were seeded in a 6-well plate and incubated in the medium without antibiotics overnight. 4T1 cells were transfected with miR-16 inhibitor (100 nM) using 4 μl of G-Fectin reagent. After 6 h, the medium was replaced with DMEM containing MSC-derived exosomes (100 μg/ml) or carrier control (PBS). After 24 h, 4T1 cells were harvested.

### Cell proliferation assay

Approximately, 2×10^3^ SVEC cells were seeded in a 96-well plate and incubated with the conditioned media from 4T1 cells that were treated with various concentrations of MSC-derived exosomes (25 μg/ml, 50 μg/ml, and 100 μg/ml) or carrier control (PBS). Cell proliferation rates were determined by an EZ-Cytox cell viability assay kit (Daeil Labservice, Seoul, Korea) according to the manufacturer's instructions.

### Transwell migration assay

The transwell migration assay was performed on SVEC cells using a Boyden chamber containing a polycarbonate filter with an 8 μm pore size (Corning Costar, Corning, NY). SVEC cells were serum-starved for 24 h in DMEM prior to initiation of the experiment. The lower chambers were filled with 600 μl of conditioned medium from 4T1 cells that were treated with various concentrations of MSC-derived exosomes (25 μg/ml, 50 μg/ml, and 100 μg/ml) or carrier control (PBS). Approximately, 7×10^4^ SVEC cells were resuspended in 200 μl of serum-free DMEM and added to the upper chamber. Cells were incubated at 37°C for 24 h to allow cell migration through the membrane. Migratory cells were fixed in 4% paraformaldehyde and stained with crystal violet. The images were captured and further analyzed using Image J software (NIH, Bethesda, MD).

### Wound healing assay

SVEC cells were seeded and cultured to 90% confluency in a 12-well plate. SVEC cells were incubated with serum-free DMEM overnight to synchronize and wounded with a 200 μl pipette tip. Then, SVEC cells were incubated in 500 μl of the conditioned media from 4T1 cells stimulated with MSC-derived exosomes (100 μg/ml) or carrier control (PBS) for 24 h. Also, neutralization of VEGF was performed adding anti-VEGF antibodies (Angio-Proteomie, Boston, MA) to the conditioned media from 4T1 cells. Wound healing was quantified as the average length of the elongation of wound edges over 24 h by using Studio Lite, version 1.0 (Better Light, Inc., San Carlos, CA).

### Tube formation assay

Matrigel (300 μl; BD Biosciences, San Jose, CA) was added to each well of a 24-well plate and allowed to polymerize. SVEC cells were serum-starved for 24 h in DMEM prior to initiation of the experiment and 2×10^4^ SVEC cells were seeded in a Matrigel-coated well. The cells were treated with conditioned media collected from 4T1 cells stimulated with MSC-derived exosomes (100 μg/ml) or carrier control (PBS) for 24 h and viewed under a microscope.

### Animal model

Animal experiments were carried out according to the guidelines of the Institutional Animal Care and Use Committee (IACUC) of Seoul National University. The experiments were performed after receiving approval from Institutional Biosafety Committee (IBC: SNUIB-P110525-1) and IACUC (SNU-110527-1). Five-week-old female BALB/c mice were purchased from SLC Japan (Shizuoka, Japan) and maintained under specific pathogen-free conditions. Mice were randomly divided into three groups and all groups received subcutaneous injections of 100 μl PBS per mouse containing 2×10^5^ 4T1 cells alone or 2×10^5^ 4T1 cells mixed with 100 μg of MSC-derived exosomes or 2×10^5^ 4T1 cells mixed with 200 μg of MSC-derived exosomes. The tumor sizes of mice in the groups were measured with a caliper three times a week from 20 days after tumor challenges and the tumor volume was calculated using the formula: 0.5×(Width)^2^×Length [29].

### Immunohistochemistry

Mice were sacrificed 5 weeks after tumor inoculation. The tumors were harvested and fixed in 10% neutral buffered formalin. Immunohistochemical analysis was performed on the paraffin-embedded tumors using the standard techniques with antibodies as follows: VEGF (Abcam, Cambridge, MA) and CD31 (Santa Cruz Biotechnology, Inc., Santa Cruz, CA) [30].

### Statistical methods

Differences were evaluated by unpaired two-tailed Student's *t* test. All experiments were performed at least three times. A value of *P*<0.05 was considered significant.

## Results

### Characterization of MSC-derived exosomes

**Figure 1:**
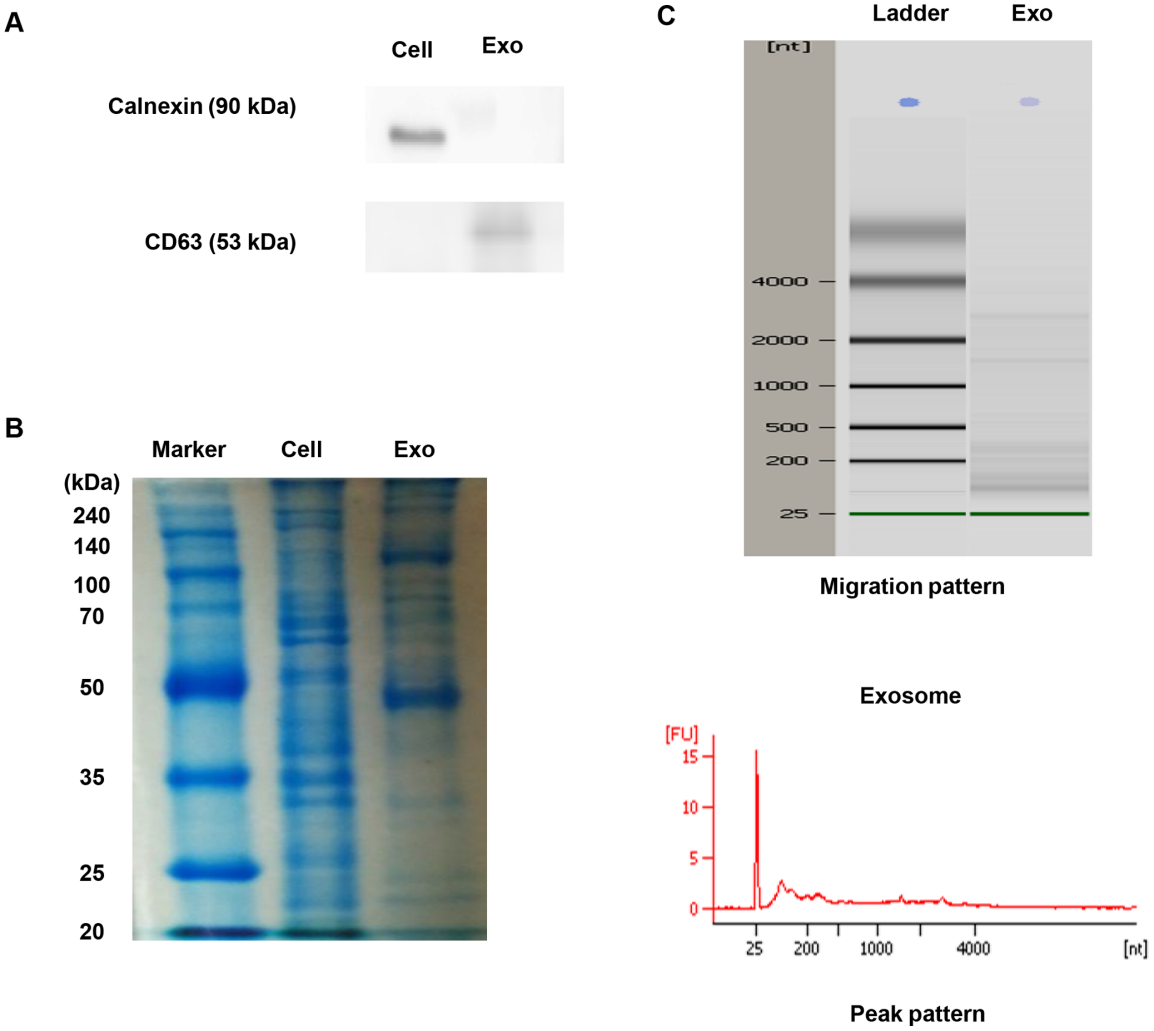
Characterization of MSC-derived exosomes. (A) Western blotting was performed with MSCs (Cell) or MSC-derived exosomes (Exo). Calnexin expression in MSCs and CD63 expression in MSC-derived exosomes were detected. (B) Protein was isolated from MSCs and MSC-derived exosomes. An equivalent amount (50 ug) of protein from MSCs and MSC-derived exosomes was loaded and run on a 10% SDS gel and stained with Coomassie blue. (C) RNA was extracted from MSC-derived exosomes and analyzed by a Bioanalyzer. Representative bioanalyzer profile of the RNA contained in MSC-derived exosomes showed that the ribosomal subunits 28S and 18S were barely detectable.

To investigate the potential effects of MSC-derived exosomes on tumor behavior, we first isolated exosomes from the culture supernatants of MSCs as described in Materials and Methods. To examine whether MSC-derived exosomes were successfully purified, we performed immunoblotting. As shown in Figure 1A, CD63 (a representative marker of exosomes) was detected in the isolated exosomes, whereas calnexin (a marker of endoplasmic reticulum) was not [26], [31]. These data demonstrated that MSC-derived exosomes were successfully purified.

Next, we compared the cellular contents in exosomes with those in their corresponding donor cells. We first evaluated the protein expression profile in MSCs and MSC-derived exosomes. Equivalent amount of proteins extracted from MSCs and MSC-derived exosomes were separated by 10% SDS-PAGE and stained with Coomassie Blue (Figure 1B). Consistent with previous reports, abundant proteins were found in exosomes and the protein isolated from exosomes had a different profile [26], [32]. We subsequently analyzed the profile of total RNAs extracted from exosomes using capillary electrophoresis (Figure 1C). Unlike total RNAs collected from cells, the ribosomal subunits 28S and 18S were scarcely detected. Instead, the majority of the total RNAs in exosomes were below 2 kb, suggesting the presence of small RNAs, such as miRNAs [7], [32]. Taken together, our results indicated that both protein and RNA contents in exosomes were different from those in the donor cells.

### Celluar uptake of MSC-derived exosomes into 4T1 cells

**Figure 2:**
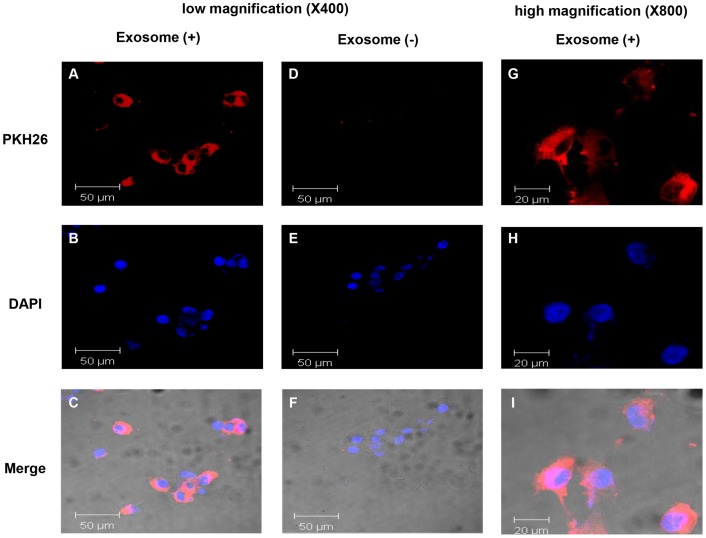
Cellular internalization of MSC-derived exosomes into 4T1 cells. 4T1 cells were incubated with 25 μg of MSC-derived exosomes that were labeled with PKH26 (red) for 24 h. 4T1 cells were also incubated with PKH26 without exosomes as a negative control to observe carryover of PKH26. Low magnification images of 4T1 cells incubated with exosomes (A–C), or negative controls without exosomes (D–F) are shown (×400). High magnification images of 4T1 cells incubated with exosomes (G–I) are shown (×800).

To study the internalization of MSC-derived exosomes by mouse breast cancer cells (4T1), MSC-derived exosomes were labeled with the fluorescent dye, PKH26, as described in Materials and Methods. PKH26-labeled exosomes were incubated with 4T1 cells for 24 h and cellular uptake of MSC-derived exosomes was observed under the confocal laser microscopy (Figure 2). We found that PKH26-labeled exosomes were localized in the cytoplasm of 4T1 cells, implying that MSC-derived exosomes can be internalized by tumor cells.

### MSC-derived exosomes down-regulate VEGF expression in 4T1 cells

**Figure 3:**
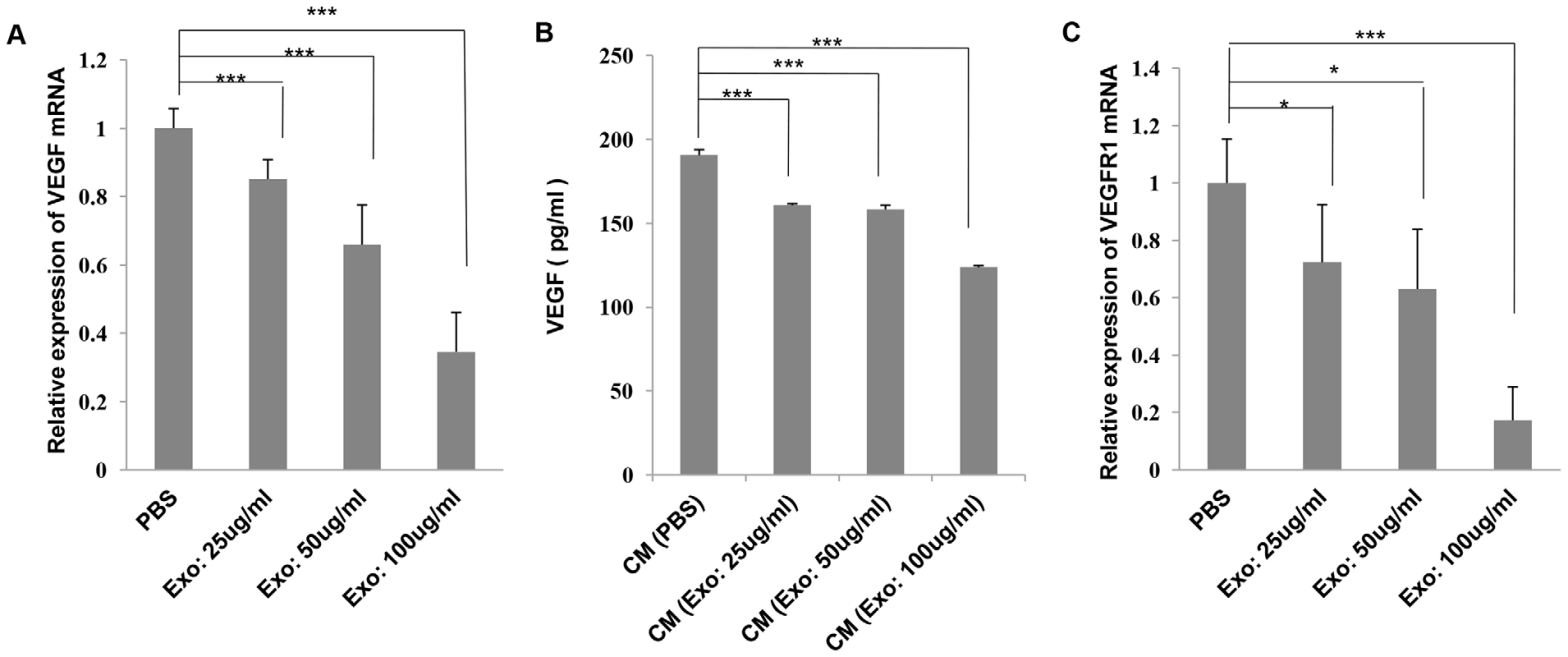
Down-regulation of VEGF expression in 4T1 cells by MSC-derived exosomes. (A) 4T1 cells were incubated with various concentrations of MSC-derived exosomes (25 μg/ml, 50 μg/ml, and 100 μg/ml) or carrier control (PBS) for 48 h. The mRNA levels of VEGF were evaluated using qRT-PCR. (B) The levels of secreted VEGF in the conditioned media from 4T1 cells that were treated with various concentrations of MSC-derived exosomes (25 μg/ml, 50 μg/ml, and 100 μg/ml) or carrier control (PBS) for 24 h were estimated by enzyme-linked immunosorbent assay. (C) The mRNA levels of VEGFR-1 in 4T1 cells that were stimulated with MSC-derived exosomes were analyzed by qRT-PCR. The values are presented as the mean ± D; n = 3 for each group. Significant differences were evaluated using an unpaired two-tailed Student's t-test. NS; Not significant, *P<0.05, **P<0.01, ***P<0.001 compared with control.

It is well-established that VEGF and its receptors, VEGFR-1 and VEGFR-2, play a crucial role in vasculogenesis and tumor angiogenesis [33], [34]. A recent study reported that exosomes derived from human bone marrow MSCs enhance tumor angiogenesis *in vivo* [35]. To analyze the angiogenic effects of exosomes derived from murine MSCs on breast cancer cells, we evaluated the mRNA levels of VEGF and its receptors in 4T1 cells that were treated with various concentrations of MSC-derived exosomes. Unexpectedly, we found that MSC-derived exosomes down-regulated the mRNA level of VEGF in 4T1 cells in a dose-dependent manner (Figure 3A). To further confirm the protein level of VEGF, we subsequently measured the amount of VEGF secreted into the conditioned media from 4T1 cells that were stimulated with MSC-derived exosomes. Addition of MSC-derived exosomes decreased VEGF secretion in 4T1 cells, which was consistent with the down-regulation of VEGF mRNA levels (Figure 3B). Furthermore, the mRNA level of VEGFR-1 in 4T1 cells was substantially decreased when 4T1 cells were co-incubated with MSC-derived exosomes (Figure 3C). However, there were no statistically significant differences in the mRNA level of VEGFR-2 between exosome-treated and control groups (data not shown). This was likely due to 4T1 cells scarcely expressing VEGFR-2 [36]. To further examine any cytotoxic effects of MSC-derived exosomes on 4T1 cells, we evaluated the proliferation and viability of 4T1 cells that were treated with various concentrations of MSC-derived exosomes for 24, 48, and 72 h. Intriguingly, the results revealed that MSC-derived exosomes had no significant effect on the proliferation of 4T1 cells at the three time-points (data not shown). Thus, we speculated that MSC-derived exosomes did not directly affect 4T1 cells, but instead might have indirect effects on 4T1 cells.

### MiR-16 suttled by MSC-derived exosomes reduces the VEGF expression in 4T1 cells

**Figure 4:**
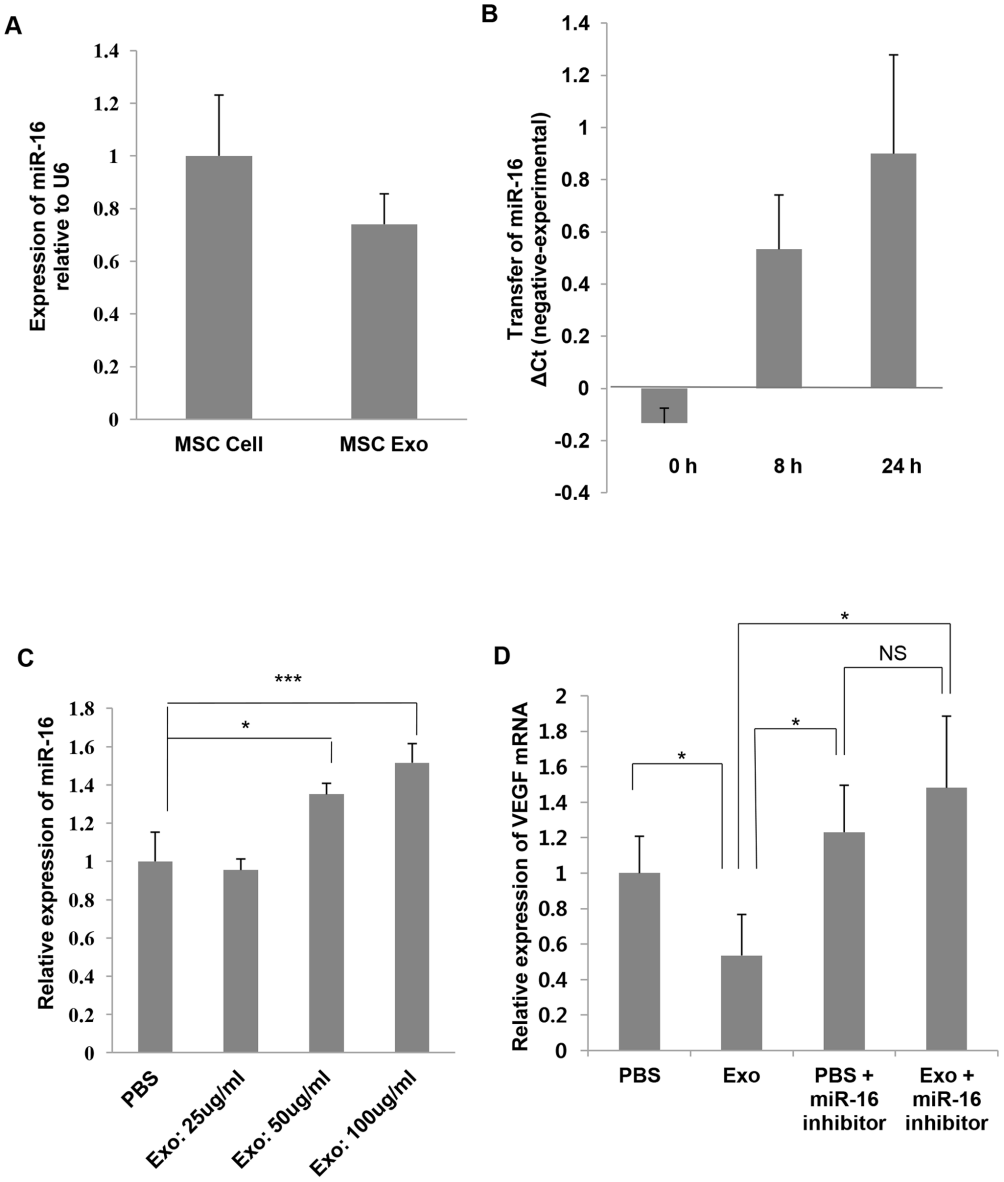
Transfer of miR-16 via MSC-derived exosomes. (A) qRT-PCR was used to measure the levels of miR-16 in MSCs and MSC-derived exosomes. (B) 4T1 cells were incubated with MSC-derived exosomes and α-amanitin (experimental sample) or α-amanitin alone (negative control). Transfer of miR-16 was determined by qRT-PCR and positive values indicate transfer of miR-16. (C) 4T1 cells were incubated with various concentrations of MSC-derived exosomes (25 μg/ml, 50 μg/ml, and 100 μg/ml) or carrier control (PBS) for 48 h and miR-16 levels were evaluated. (D) 4T1 cells were transfected with miR-16 inhibitor and incubated with MSC-derived exosomes (100 μg/ml) or carrier control (PBS) for 24 h. qRT-PCR was conducted to evaluate the VEGF mRNA expression. The values are presented as the mean ± SD; n = 3 for each group. Significant differences were evaluated using an unpaired two-tailed Student's t-test. NS; Not significant, *P<0.05, **P<0.01, ***P<0.001 compared with control.

Based on the previous observations that miRNAs are enriched in exosomes, we hypothesized that miRNAs shuttled by MSC-derived exosomes might be responsible for the down-regulation of VEGF expression in 4T1 cells. To determine candidate miRNAs, we searched the literature and various databases. It has been demonstrated that miR-16 controls the expression of VEGF [37], [38], [39]. Thus, we first examined miR-16 expression levels in MSCs and MSC-derived exosomes by performing qRT-PCR (Figure 4A). Although we used U6 snRNA as a normalization control, it should be noted that there is no normalizer known to be expressed with the same copy number in both exosomes and their corresponding donor cells [26], [32]. Therefore, we could not directly compare miR-16 expression levels in MSCs and MSC-derived exosomes. Instead, we concluded that miR-16 was co-expressed by both MSCs and MSC-derived exosomes. Next, we investigated whether MSC-derived exosomes transferred miR-16 to 4T1 cells. 4T1 cells were co-incubated with MSC-derived exosomes in the presence of α-amanitin or α-amanitin alone to suppress transcriptional activation caused by exosomes [28] and then miR-16 expression levels were evaluated using qRT-PCR (Figure 4B). The difference in Ct values between the negative control (α-amanitin alone) and experimental sample (MSC-derived exosomes and α-amanitin) was measured at the indicated times [32]. We found that the abundance of miR-16 increased gradually, suggesting transfer of miR-16 from exosomes to breast cancer cells. In addition, miR-16 expression levels in 4T1 cells that were stimulated with various concentrations of MSC-derived exosomes were inversely correlated with the levels of VEGF expression, suggesting that miR-16 transferred by MSC-derived exosomes may target VEGF (Figure 4C). To further confirm that miR-16 shuttled by MSC-derived exosomes might be associated with the down-regulation of VEGF, application of miR-16 inhibitor was conducted. 4T1 cells were transfected with miR-16 inhibitor (100 nM) and treated with MSC-derived exosomes (100 μg/ml) or carrier control (PBS) for 24 h. Intriguingly, the decreased mRNA level of VEGF in 4T1 cells by MSC-derived exosomes was rescued when miR-16 inhibitor was added (Figure 4D). Taken together, our data demonstrated that miR-16 transferred by MSC-derived exosomes reduced the VEGF expression in 4T1 cells.

### MSC-derived exosomes suppress angiogenesis *in vitro*

**Figure 5:**
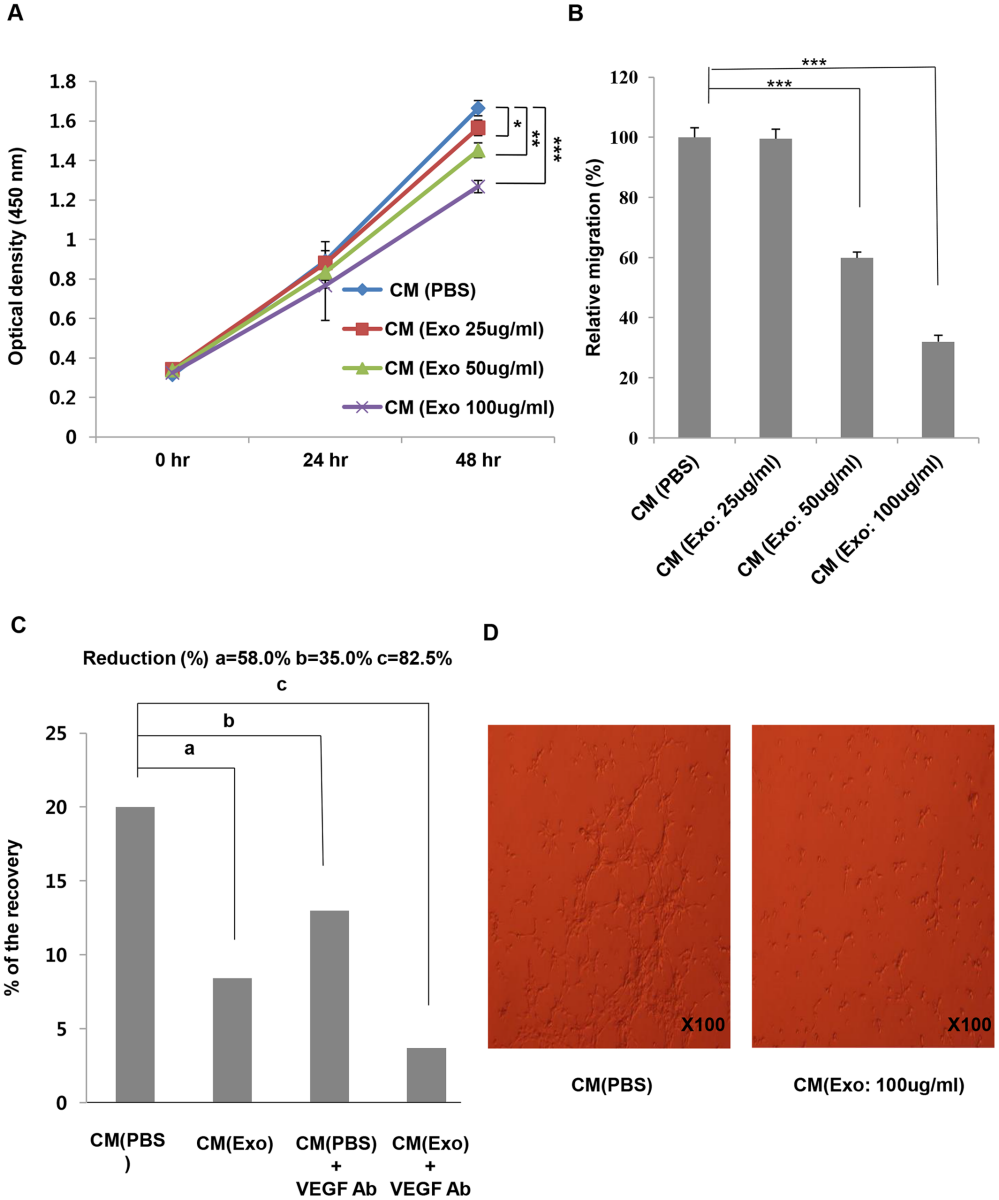
MSC-derived exosomes inhibit proliferation and migration of SVEC cells *in vitro*. (A) 2.0×10^3^ SVEC cells were incubated with the conditioned media from 4T1 cells that were treated with various concentrations of MSC-derived exosomes or carrier control (PBS). Cell proliferation rates were determined by an EZ-Cytox cell viability assay kit. (B) SVEC cells transwell migration assay was performed in the presence of the conditioned media from 4T1 cells that were treated with various concentrations of MSC-derived exosomes or carrier control (PBS) in the lower chambers. Serum-starved SVEC cells were added to the upper chamber and incubated for 24 h to allow cell migration through the membrane. The membranes were stained with crystal violet and cell migration was analyzed by Image J. (C) SVEC cells were scratched and incubated with the conditioned media from 4T1 cells stimulated with MSC-derived exosomes (100 μg/ml) or vehicle control (PBS) for 24 h. In order to neutralize VEGF derived from 4T1 cells, anti-VEGF antibodies (20 μg/ml) were added to the conditioned media. Photographs were taken immediately and 24 h after wounding (data not shown) and analyzed by Studio Lite, version 1.0. (D) SVEC cells were serum-starved for 24 h and 2×10^4^ SVEC cells were seeded in a Matrigel-coated well. The cells were treated with conditioned media collected from 4T1 cells stimulated with MSC-derived exosomes (100 μg/ml) or carrier control (PBS) for 24 h and viewed under a microscope. The values are presented as the mean ± SD; n = 3 for each group. Significant differences were evaluated using an unpaired two-tailed Student's t-test. *P<0.05, **P<0.01, ***P<0.001 compared with control.

Previous studies have demonstrated that VEGF, a potent angiogenic factor, is overexpressed in various cancer types [20], [40]. Moreover, it has been reported that VEGF is a key regulator of endothelial cell (EC) proliferation and migration [41], [42]. To determine whether the decreased amount of VEGF by MSC-derived exosomes had effects on EC proliferation and migration, we prepared the conditioned media from 4T1 cells that were treated with various concentrations of MSC-derived exosomes and SVEC cells, murine endothelial cell line [43]. We then performed SVEC cells proliferation assay and transwell migration assay by treating SVEC cells with the conditioned media from 4T1 cells. The conditioned media from 4T1 cells treated with MSC-derived exosomes significantly decreased the rate of proliferation and migration of SVEC cells compared to the conditioned media from untreated 4T1 cells (Figure 5A and 5B). As a further check, we next performed a wound healing assay. The conditioned media from 4T1 cells stimulated with MSC-derived exosomes (100 μg/ml) were collected and transferred to SVEC cells. In this experiment, we used a VEGF neutralizing antibody to verify that VEGF from 4T1 cells plays a significant role in SVEC cells migration. Consistent with the transwell migration assay, the decreased amount of VEGF from 4T1 cells caused by MSC-derived exosomes resulted in the reduction in SVEC cells migration. Furthermore, the incubation with a VEGF neutralizing antibody led to even more decreases in SVEC cells migration, demonstrating that VEGF derived from 4T1 cells is a crucial factor to SVEC cells angiogenesis (Figure 5C). To evaluate the effect of MSC-derived exosomes on an ability of endothelial cells to form vessel-like structures, a tube formation assay was conducted on Matrigel-coated wells. There were significant decreases in tube formation in the group treated with the conditioned media from exosome-stimulated 4T1 cells (Figure 5D). Taken together, our data showed that the change in VEGF secretion by MSC-derived exosomes caused the lower rate of EC proliferation and migration *in vitro*.

### MSC-derived exosomes inhibit tumor growth and angiogenesis *in vivo*

**Figure 6:**
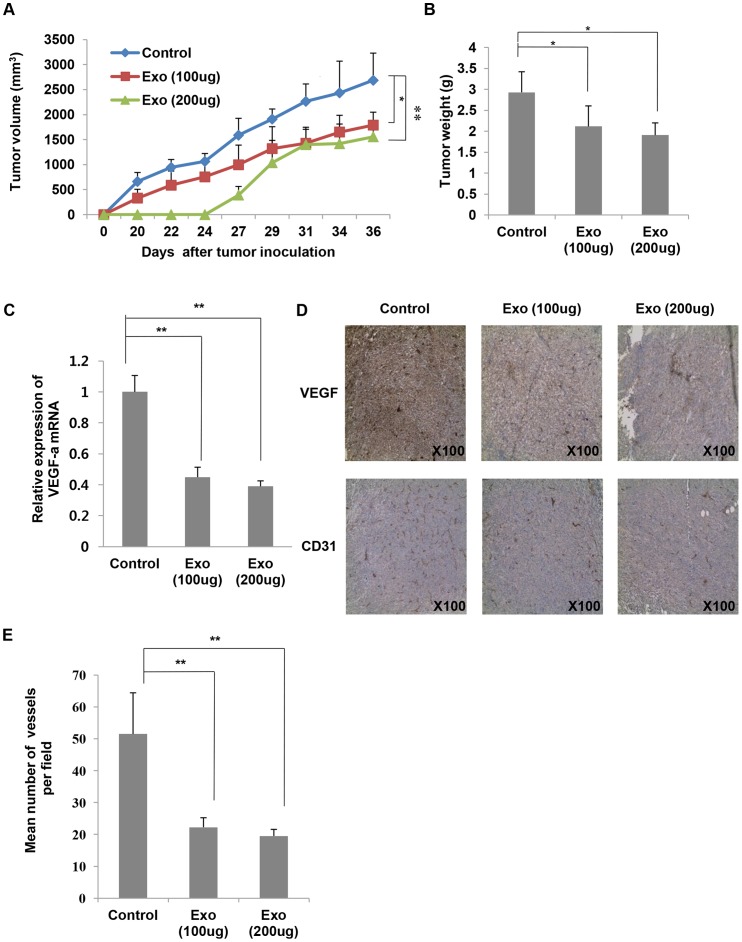
MSC-derived exosomes suppress angiogenesis *in vivo*. (A) BALB/c mice received subcutaneous injections of 100 μl PBS per mouse containing 2×10^5^ 4T1 cells alone or 2×10^5^ 4T1 cells mixed with 100 μg of MSC-derived exosomes or 2×10^5^ 4T1 cells mixed with 200 μg of MSC-derived exosomes. The tumor sizes of mice in the groups were measured with a caliper three times a week from 20 days after tumor challenges. (B) The tumor weight was measured. (C) VEGF mRNA expressions in tumor tissues were analyzed by using qRT-PCR. (D) Immunohistochemical features of the tumor tissues were shown. Formalin-fixed paraffin sections were stained with anti-VEGF and anti-CD31 antibodies. (E) The mean number of vessels in tumor histologic sections was quantified. The values are presented as the mean ± SD; n = 4 for each group. Significant differences were evaluated using an unpaired two-tailed Student's t-test. *P<0.05, **P<0.01, ***P<0.001 compared with control.

To further assess the role of MSC-derived exosomes in tumor growth and angiogenic activity *in vivo*, we established tumor models in 5-week-old female BALB/c mice by subcutaneously injecting 4T1 cells alone or 4T1 cells mixed with MSC-derived exosomes. Tumor sizes were measured three times a week and the observations lasted over 36 days after tumor challenges. Tumor growth in the exosome co-implantation group was significantly inhibited (Figure 6A). Furthermore, tumor growth in the 200 μg of exosome co-implantation group was more effectively suppressed, suggesting that MSC-derived exosomes have anti-tumor effects *in vivo*. Tumor weight was also measured after sacrifice. There were statistically significant differences in tumor weight between the exosome co-implantation group and control group (Figure 6B).

Next, we evaluated the effects of MSC-derived exosomes on tumor angiogenesis. Consistent with *in vitro* results, the mRNA level of VEGF was decreased in tumors from the exosome co-implantation group (Figure 6C). Furthermore, immunohistochemical analysis of tumor tissues from the three groups showed that MSC-derived exosomes greatly inhibited tumor angiogenesis (Figure 6D). Tumor histologic sections from the exosome co-implantation group exhibited relatively weak expression of VEGF and CD31 (a marker of vascular endothelial cells) compared to those from the control mice. Analysis of histologic sections indicated that the mean number of vascular structures per filed was lower in the exosomes-treated than untreated tumors (Figure 6E). As a result, these observations suggest that MSC-derived exosomes effectively suppress angiogenesis *in vivo*.

## Discussion

Cell-to-cell communication is a dynamic mechanism that enables normal cellular activities and maintains tissue homeostasis. Recent studies have demonstrated that exosomes released by different cell types may act as a mediator of cell-to-cell communication. Exosomes contain genetic materials under the form of mRNAs and miRNAs, thus allowing exchange of information between cells [4]. A number of cell types including tumor cells can epigenetically reprogram their neighboring cells by releasing exosomes [44]. A recent study suggested that hepatocellular carcinoma cell (HCC)-derived exosomes contained a set of miRNAs, modulating the function of recipient HCC cells [26].

Tumors are complex tissues that include various types of cells such as mesenchymal cells, immune cells, and vascular endothelial cells. Therefore, the interaction between cancer cells and their microenvironment has been extensively studied. Over the past 10 years, MSCs have been the subject of a growing interest owing to their ability to home at injury sites such as inflammation and neoplasia [45]. Once they are incorporated into tumors, they exert complex effects on tumors. Whether MSCs are pro- or anti-tumorigenic has been a subject of controversy. Interestingly, some investigators reported that MSCs promote tumor growth, whereas other groups demonstrated that MSCs suppress tumor progression [18]. Even though researchers proposed various mechanisms, the effects of MSC-derived exosomes on tumor cells remain to be investigated.

The aim of this study was to assess the effects of MSC-derived exosoems on tumor behavior, especially in respect to angiogenesis. We successfully isolated exosomes from the culture supernatants of murine bone marrow MSCs. We observed that MSC-derived exosomes were similar to those from other cells in terms of molecular contents [26], [31]. We used a bioanalyzer to examine the profile of total RNA extracted from exosomes. Our results demonstrated that MSC-derived exosomes contained small RNAs of the size of miRNAs, suggesting that miRNAs shuttled by MSC-derived exosomes might alter the function of recipient cells in a paracrine manner.

A recent study reported that exosomes from human bone marrow MSCs increase VEGF in tumors, resulting in enhanced tumor angiogenesis *in vivo* [35]. Thus, we first investigated whether MSC-derived exosomes had a similar effect on VEGF expression in our settings. Surprisingly, the results indicated that MSC-derived exosomes down-regulated the mRNA and protein levels of VEGF in tumor cells in a concentration-dependent manner. This inconsistency might be explained by different tumor types, *in vivo* tumor models, and the heterogeneity in MSCs [18]. Furthermore, we observed that MSC-derived exosomes do not exert significant effects on tumor cell proliferation and viability. These findings suggested that MSC-derived exosomes might indirectly alter tumor cell behavior.

To further study the molecular mechanisms underlying the decrease in VEGF of tumor cells by MSC-derived exosomes, we paid attention to previous studies that miR-16 can down-regulate the expression of VEGF [38], [39]. Thus, we evaluated miR-16 expression levels in MSCs and MSC-derived exosomes. Our data showed that miR-16 was co-expressed by both MSCs and MSC-derived exosomes and MSC-derived exosomes transferred miR-16 into tumor cells. Additionally, miR-16 levels were inversely related to VEGF levels in tumor cells that were treated with MSC-derived exosomes. Application of miR-16 inhibitor confirmed that the exosome-derived miR-16 reduced the expression of VEGF in 4T1 cells.

We next examined whether the decreased amount of VEGF by MSC-derived exosomes affected endothelial cell proliferation and migration. Our data indicated that the conditioned media from tumor cells treated with MSC-derived exosomes significantly inhibited the proliferation and migration of endothelial cells, suggesting that MSC-derived exosomes suppress angiogenesis *in vitro*.

We further studied the effects of MSC-derived exosomes on tumor growth and angiogenesis *in vivo.* Consistent with *in vitro* observations, our data clearly exhibited that MSC-derived exosomes inhibited VEGF expression *in vivo*. It is noteworthy that tumor growth in the 200 μg of exosome co-implantation group increased abruptly. We speculated that although the high concentration of MSC-derived exosomes effectively suppressed tumor growth and angiogenesis in the beginning, anti-tumor effects of MSC-derived exosomes were weakened over time.

In conclusion, the results of the present study demonstrate that MSC-derived exosomes suppress angiogenesis and tumor progression by inhibiting the expression of VEGF in tumors *in vitro* and *in vivo*. Also, miR-16 shuttled by MSC-derived exosomes is partially responsible for the down-regulation of VEGF in tumor cells. Thus, our findings support the proposal that MSC-derived exosomes can be an effective anti-angiogenetic agent for anti-tumor therapy. To the best of our knowledge, this is the first report to suggest that MSC-derived exosomes may epigenetically reprogram the function of tumor cells by transferring anti-angiogenetic miRNAs.
